# A21 EFFECTS OF LYOPHILIZED FECAL FILTRATE COMPARED TO LYOPHILIZED DONOR STOOL IN THE TREATMENT OF RECURRENT CLOSTRIDIOIDES DIFFICILE INFECTION: A MULTI-CENTER RANDOMIZED TRIAL

**DOI:** 10.1093/jcag/gwad061.021

**Published:** 2024-02-14

**Authors:** D Kao, K Wong, C Lee, T Steiner, R Franz, C McDougall, M Silva, M Yaskina, J Walter, V Loo, R Loebenberg, H Xu, T Louie

**Affiliations:** University of Alberta College of Health Sciences, Edmonton, AB, Canada; University of Alberta College of Health Sciences, Edmonton, AB, Canada; UBC, Victoria, BC, Canada; UBC, Victoria, BC, Canada; University of Alberta College of Health Sciences, Edmonton, AB, Canada; University of Alberta College of Health Sciences, Edmonton, AB, Canada; University of Calgary Cumming School of Medicine, Calgary, AB, Canada; University of Alberta, Edmonton, AB, Canada; University College Cork, Cork, Cork, Ireland; McGill University, Montreal, QC, Canada; University of Alberta, Edmonton, AB, Canada; Indiana University, Bloomington, IN; University of Calgary Cumming School of Medicine, Calgary, AB, Canada

## Abstract

**Background:**

Fecal microbiota transplantation (FMT) is the most effective therapy in the treatment of recurrent *Clostridioides difficile* infection (rCDI), and can be offered in fresh, frozen or lyophilized formulation with similar efficacy. Microbial engraftment is thought to be one of the mechanisms mediating its efficacy. However, the importance of microbes has been questioned by two small preliminary clinical studies that showed sterile fecal filtrate could prevent *C. difficile* recurrence.

**Aims:**

To establish whether lyophilized sterile fecal filtrate (LSFF) is noninferior to lyophilized FMT (LFMT) in efficacy

**Methods:**

In this double-blind, non-inferiority study conducted in 4 academic centers in Alberta and British Columbia, we randomized rCDI patients with ≥ 3 CDI episodes to either LSFF or LFMT at a 1:1 ratio between 2019 and 2023.The non inferiority margin was 10%. Each treatment dose consisted of 15 oral capsules. Participants who failed the assigned treatments were offered open-label LFMT. An interim analysis was planned after reaching 50% of recruitment target (124/248 participants). Primary outcome was the proportion of participants without CDI recurrence 8 weeks following assigned treatment. Secondary outcomes included 1) the proportion of participants without CDI recurrence 24 weeks following assigned treatment, 2) serious adverse events (mortality, hospitalization and infections related to treatments), and 3) minor adverse events (nausea, vomiting, abdominal pain and fever). Exploratory outcomes included changes in quality of life, stool microbial composition analyses, as well as patient preferences.

**Results:**

137 participants were randomized (mean [SD] age, 61.2 [18.6] years; 89 women [65.4%]; mean CDI episodes 3.8) to LFMT group (66 participants) and LSFF (71 participants) group. In per-protocol analysis, prevention of rCDI 8 weeks after assigned treatment was achieved in 63.8% of participants in LSFF group and 86.9% in LFMT group (difference, -23.1%), resulting in trial termination. Serious adverse events were infrequent: 4 hospitalizations deemed not related and 1 hospitalization possibly related, and 1 death from COVID deemed not related to assigned treatment. Minor adverse events were self-limited and infrequent: nausea (6), vomiting (2), abdominal discomfort (15) and fever (2).

**Conclusions:**

Among adults with rCDI, LSFF was inferior to LFMT by oral capsules in preventing recurrent infection over 8 weeks. The result emphasizes the importance of microbes in mediating FMT efficacy. Preserving microbial viability in future development of microbial therapeutics is paramount.

**Funding Agencies:**

CIHR

